# Lack of sex chromosome specific meiotic silencing in platypus reveals origin of MSCI in therian mammals

**DOI:** 10.1186/s12915-015-0215-4

**Published:** 2015-12-10

**Authors:** Tasman J. Daish, Aaron E. Casey, Frank Grutzner

**Affiliations:** The Robinson Research Institute, School of Biological Sciences, University of Adelaide, Adelaide, 5005 South Australia Australia

**Keywords:** Meiosis, Meiotic sex chromosome inactivation, Meiotic silencing, Monotremes, Genetics

## Abstract

**Background:**

In therian mammals heteromorphic sex chromosomes are subject to meiotic sex chromosome inactivation (MSCI) during meiotic prophase I while the autosomes maintain transcriptional activity. The evolution of this sex chromosome silencing is thought to result in retroposition of genes required in spermatogenesis from the sex chromosomes to autosomes. In birds sex chromosome specific silencing appears to be absent and global transcriptional reductions occur through pachytene and sex chromosome-derived autosomal retrogenes are lacking. Egg laying monotremes are the most basal mammalian lineage, feature a complex and highly differentiated XY sex chromosome system with homology to the avian sex chromosomes, and also lack autosomal retrogenes. In order to delineate the point of origin of sex chromosome specific silencing in mammals we investigated whether MSCI exists in platypus.

**Results:**

Our results show that platypus sex chromosomes display only partial or transient colocalisation with a repressive histone variant linked to therian sex chromosome silencing and surprisingly lack a hallmark MSCI epigenetic signature present in other mammals. Remarkably, platypus instead feature an avian like period of general low level transcription through prophase I with the sex chromosomes and the future mammalian X maintaining association with a nucleolus-like structure.

**Conclusions:**

Our work demonstrates for the first time that in mammals meiotic silencing of sex chromosomes evolved after the divergence of monotremes presumably as a result of the differentiation of the therian XY sex chromosomes. We provide a novel evolutionary scenario on how the future therian X chromosome commenced the trajectory toward MSCI.

**Electronic supplementary material:**

The online version of this article (doi:10.1186/s12915-015-0215-4) contains supplementary material, which is available to authorized users.

## Background

The evolution of heteromorphic sex chromosomes conflicted with checkpoints regulating meiotic progression following unpaired DNA detection. Eutherian sex chromosome pairing is restricted to the pseudoautosomal region (PAR), consequently leaving the majority of the sex chromosome DNA unpaired through meiotic prophase [1]. Numerous strategies have independently emerged through evolution to deal with the presence of heterologous sequences during meiotic synapsis revealing the dynamic and adaptive nature of regulatory mechanisms in the germline in response to sex chromosome divergence [2].

The hallmark feature of meiotic pachytene cells in male mammals is the transcriptional silencing of genes residing on unpaired sex chromosome DNA, termed Meiotic Sex Chromosome Inactivation (MSCI) [3]. This response silences unpaired sequences by utilising DNA damage repair (DDR) pathway components and recruiting chromatin remodelling factors [4, 5]. In response to silencing, retrotransposition to autosomes of X linked genes essential for meiotic progression enable expression maintenance over the period of meiotic silencing [6, 7]. Therian MSCI is postulated to prevent the exchange of genetic material between heterologous sequences or enable checkpoint avoidance [8, 9].

Variations on the MSCI theme are present in diverse species spanning large evolutionary timeframes such as fungi, nematode, insects, birds and mammals such as opossum, mouse and human. However, there are fundamental differences in the mode by which the sex linked genes are repressed. For example, mammals and Nematode repress meiotic expression by direct epigenetic modification while *Neurospora crassa* achieves meiotic silencing post-transcriptionally [10] and recent reports have disputed the presence of MSCI in *Drosophila* and chicken [11, 12]. Clear distinctions are also present in the manner by which sex chromosomes associate through meiosis. In mouse and human, the XY mediate pairing initially by PAR synapsis, the marsupial XY, which lacks a PAR, is tethered to a dense plate structure [13, 14], and the female chicken ZW undergoes complete pseudosynapsis [15].

Monotremes are key to understanding the evolution of MSCI in mammals. Their sex chromosomes have homology to the chicken Z and chromosome 6 is homologous to the future therian X chromosome, however the heterogametic sex, unlike chicken, is male [16, 17]. Also, platypuses have a complex 5X and 5Y sex chromosome system which pair to form a chain during prophase I in preparation for alternate XY segregation [18–20]. Thus, determining the existence of monotreme MSCI may not only reveal potentially novel meiotic silencing mechanisms but also pinpoint when MSCI evolved in mammals.

In this study we sought to determine whether MSCI exists in platypus using DNA fluorescence in situ hybridisation (FISH), immunohistochemistry and expression analyses to characterise the epigenetic and sex chromosome linked gene activity through prophase I. Surprisingly, unlike other mammals, platypus prophase I nuclei maintain a schedule of low general transcription and lack hallmark epigenetic MSCI modifications on sex chromosomes. In addition, we also saw similarities with chicken regarding the nature of heterologous sex chromosome self-association but also therian-like nucleolar association. This study reveals avian and mammalian aspects of sex chromosome meiotic dynamics in platypus representing the transition to sex chromosome specific silencing arising early in mammalian evolution possibly by the co-opting of nucleolar associated repressive machinery and the different gene sets on the therian X being indispensable for meiotic progression.

## Results

### Platypus sex chromosomes form a condensed body at pachytene

**Fig. 1:**
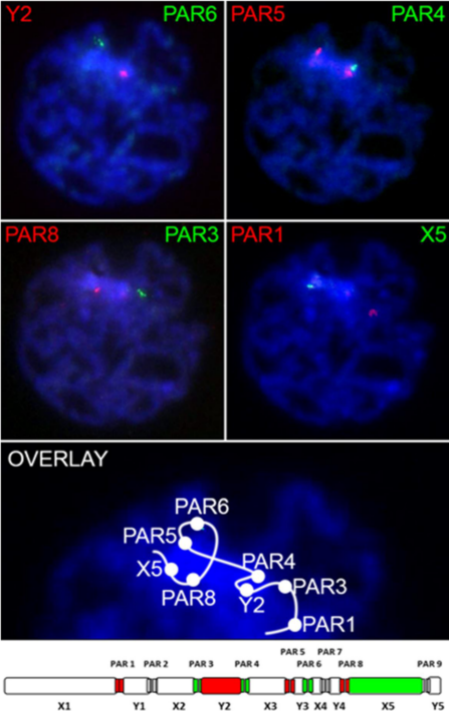
Sex chromosome chain conformation at late prophase I. Total testis cell suspensions were methanol:acetic acid fixed prior to serial BAC probe DNA FISH hybridisations. Dual colour DNA FISH signals were recorded prior to additional dual colour FISH experiments on the same slide. All signals are positioned within or immediately adjacent a DAPI intense heterochromatic mass. *Bottom panel* shows representative positions of collated signals from four experiments using eight different BAC probes linked by an arbitrarily drawn line. Schematic at *bottom* is of sex chromosome chain with corresponding positions of BAC probes. *FISH* fluorescence in situ hybridisation, *DAPI* 4’6-diamidino-2-phenylindole

To assess sex chromosome distributions and chromatin compaction status during prophase I we prepared methanol:acetic acid fixed total testis suspensions and used serial DNA FISH with sex chromosome specific BAC probes. Cells in prophase with condensed chromatin elements representing chromosomes undergoing synapsis were consistently observed to feature a distinct 4’6-diamidino-2-phenylindole (DAPI) intense mass (Fig. 1). All DNA FISH probes either targeting PARs or sex chromosomes co-localised with the DAPI intense mass indicating its primary composition is sex chromatin.

**Fig. 2:**
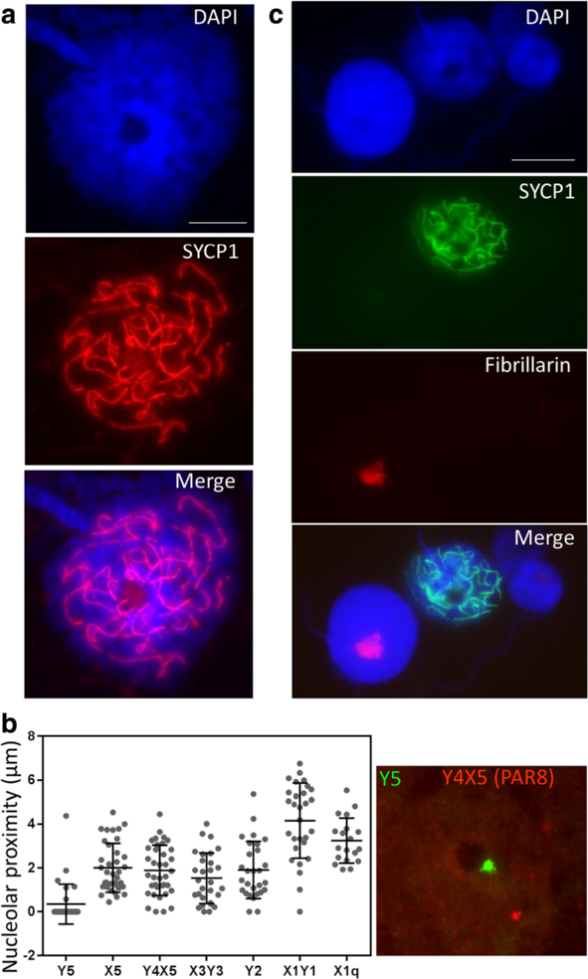
Nucleolar activity and sex chromosome proximity in SYCP1 positive nuclei. Surface spreads of PFA fixed total testis suspensions were immunostained for SYCP1 or SYCP1 and fibrillarin prior to DNA FISH (SYCP1 slides only). **a** large DAPI void predicted to be the nucleolus was present in all nuclei with advanced stages of synapsis (pachynema) shown by SYCP1 staining. We identify this structure as the nucleolar like body (NLB). **b** DNA FISH signal positions were measured in late prophase I spermatocytes based on proximity to the NLB. An ordered hierarchy of proximity was observed with Y5 having consistent close association and the X1 end of the chain displaying more variable positions. The number of nuclei for each sex chromosome element analysed was as follows: X1q 19, X1Y1 (PAR1) 29, Y2 28, X3Y3 (PAR5) 27, Y4X5 (PAR8) 34, X5 37 and Y5 29. Graph was generated using Prism version 6. Error bars indicate mean +/− standard deviation. *Right panel* in C shows Y5 DNA FISH signal positioned against the NLB. **c** Surface spreads were immunostained for the nucleolar marker fibrillarin and SYCP1 to assess rRNA biogenesis activity at late prophase I. Fibrillarin staining is absent in late prophase I nuclei staged using SYCP1. Scale bars = 10 μm. *PFA* paraformaldehyde, *FISH* fluorescence in situ hybridisation, *DAPI* 4’6-diamidino-2-phenylindole

To visualise synapsis we then prepared surface spreads of paraformaldehyde (PFA) fixed total testis suspensions for immunostaining with an antibody against the central element component SYCP1 as no antibodies against the lateral element component SYCP3 yielded any positive staining in platypus meiotic cells. In addition, antibodies against Hormad 1/2, TopBP1, ATR, Rad51, and MDC1 were tested on platypus meiotic spreads without success by us and other investigators. All SYCP1 positive nuclei with staining patterns characteristic of advanced synapsis showed a consistent and obvious presence of a singular large circular DAPI void we describe as the nucleolar like body (NLB) (Fig. 2a). Platypus have a single nucleolar organising region (NOR) located on chromosome 6 which would result in a single nucleolus. We next used sex chromosome specific BAC probes for DNA FISH to determine the distribution of chain elements in SYCP1 positive meiotic nuclei relative to the NLB as the nucleolus is transiently associated with the silenced XY sex body in mouse as well as being required for establishing X chromosome inactivation [21–24]. We used BAC probes against X1q, PAR1, Y2, PAR5, PAR8, X5, and Y5 sex chromosome regions to visualise the proximity of individual elements to the nucleolus. Distance of the DNA FISH signals from the nucleolus for each BAC was measured in multiple SYCP1 positive meiotic nuclei to assess average nucleolar proximities (Fig. 2b). We observed that Y5 is commonly tethered to the nucleolus at pachytene (Fig. 2b right panel) and the remaining sex chromosomes generally becoming more spatially distributed toward the X1 end of the chain. Multiple sex chromosome regions showed close nucleolar proximity similar to Y5 suggesting that a transient association of the majority of the chain occurred at some stage during pachytene.

To assess nucleolar activity, we used an antibody to Fibrillarin, an important nucleolar protein with predicted methtransferase activity [25]. Fibrillarin functions in snoRNP complexes to effect rRNA modifications essential for rRNA folding and stability during their biogenesis and the presence of which indicates nucleolar activity [26]. To identify advanced prophase I spermatoctyes and assess ribosomal activity we used anti-SYCP1 in combination with anti-Fibrillarin. We observed Fibrillarin-positive nucleoli in meiotic cells not undergoing synapsis and a loss of Fibrillarin in meiotic prophase cells with advanced stages of synapsis (Fig. 2c). This indicates active ribosomal RNA biogenesis had ceased prior to its association with the sex chromosomes.

### Platypus spermatocytes in advanced prophase I have para-NLB repressive histone accumulations

There is currently no evidence to suggest MSCI occurs in monotremes. Our observation that sex chromosomes coalesce into a dense mass during prophase I and associate with the nucleolus led us to ask whether repressive histone modifications and variants are recruited to platypus sex chromatin during prophase I as observed in other mammals.

**Fig. 3:**
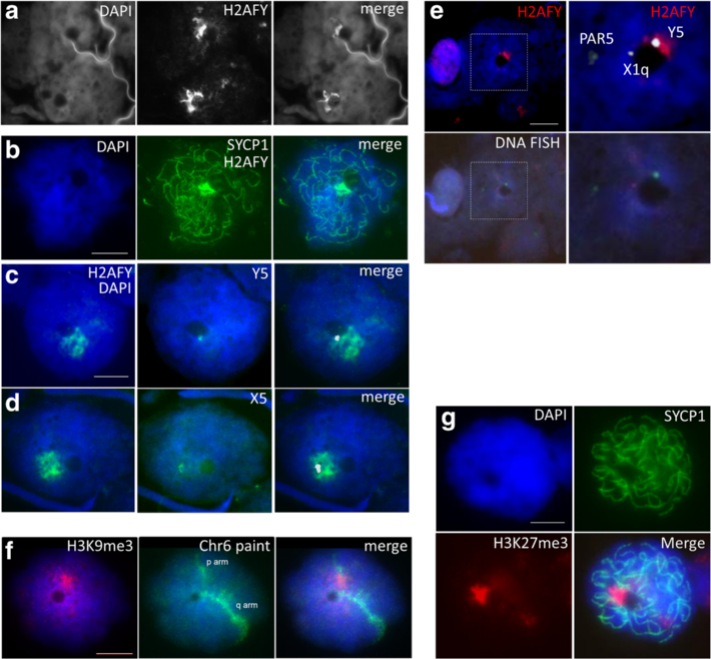
Epigenetic marks in platypus spermatocytes. Platypus total testis suspensions were PFA fixed for surface spreading and immunostained using antibodies against repressive histone marks H2AFY (panels **a**-**d** and **e**), H3K9me3 **f**, H3K27me3 **g**, followed by sex chromosome specific BAC **e** or chromosome 6 paint **f** DNA FISH. Late prophase I nuclei show variable H2AFY staining patterns positioned adjacent the NLB and restricted to SYCP1 positive nuclei **b**. H2AFY configurations include dispersed fibrillar arms **a**, more restricted foci **e**, and larger para-nucleolar blocks **c** and **d**. Y5 **c** and X5 **d** were frequently observed to co-localise with H2AFY while other elements were not observed to colocalise with H2AFY **e**. To merge DNA FISH signals with H2AFY panels the background image (non FISH signal) was subtracted and colour changed as both immunostaining and FISH probes were Alexa-488 tagged. FISH signal image adjustment was performed using standard PowerPoint tools. **f** Chromosome 6 maintains attachment to the NLB with regions of the p arm where the rDNA resides colocalising with H3K9me3. **g** H3K27me3 staining occurs in SYCP1 positive nuclei close to the NLB. Scale bars = 10 μm. *PFA* paraformaldehyde, *FISH* fluorescence in situ hybridisation

Using immunostaining on surface spreads of PFA fixed meiotic suspensions we detected strong paranucleolar enrichments for H2AFY, H3K9me3, and H3K27me3 (Fig. 3a, f and g). The most marked H3K27me3 and H2AFY paranucleolar accumulations were restricted to SYCP1 positive nuclei (Fig. 3b and g). The large histone variant H2AFY is recruited to the inactive sex body and the inactive X in mammals [27–29] so we tested whether this repressive histone variant was recruited specifically to platypus sex chromosomes which would indicate transcriptional silencing. We observed H2AFY configurations ranging from fibrillar radiations (Fig. 3a) to larger paranucleolar blocks (Fig. 3c and d). As Y5 and X5 have consistent paranucleolar positions we expected H2AFY to be coincident with these regions of the sex chromatin. Surprisingly, although these elements were observed within the H2AFY foci (Fig. 3c and d), these and additional regions of the chain such as PAR5 and X1 were also observed outside the H2AFY positive domain (Fig. 3e). For example, the frequency of paranucleolar H2AFY colocalisation with selected sex chromosomes was 31.5 % (n = 165).

We used a DNA paint FISH probe for chromosome 6 to determine whether the single NOR on chromosome 6 loses association with the nucleolus during prophase I as observed in mouse [21]. We observed chromosome 6 to retain nucleolar association in prophase I (Fig. 3f) when rRNA biogenesis had ceased as evidenced by loss of Fibrillarin staining. Interestingly we observed an association of the repetitive rDNA region of chromosome 6 with the repressive histone modification H3K9me3. A region of chromosome 6 in close proximity to the nucleolus has an absence of probe hybridisation due to depletion of repeated rDNA probe sequences on the chromosome 6 p arm. We speculate this repressive mark which overlays the repeat region is involved in preventing rDNA transcription.

### Absence of prophase I phosphorylation of histone H2AFX on autosomes or sex chromosomes

A hallmark epigenetic signature of MSCI is phosphorylation of the histone variant H2AFX (γH2AFX) on sex chromatin. Surprisingly, we could not detect this histone modification at any stage of prophase or in any meiotic cell type on surface spreads or Western blotting using cell lysates or extracted histones from total testis. Thus, neither the first or second wave of H2AFX modification normally occurring during leptotene followed by sex chromosome specific enrichment during pachytene, respectively, could be detected using multiple commercially available antibodies which could detect mouse meiotic H2AFX in our hands. Sequence analysis of platypus H2AFX shows conservation of the phosphorylation site at serine 139. To consider technical reasons for the lack of detection in platypus we asked whether we could detect γH2AFX in cultured platypus fibroblast cells following DSB induction by gamma irradiation. Following a 5 Grey exposure we detected H2AFX phosphorylation in both mouse and platypus fibroblasts (Additional file 1: Figure S1) demonstrating the antibody could detect the epitope using our methods. We were also able to detect the repair pathway sensor kinase ATM at induced γH2AFX foci in fibroblasts indicating a DNA damage response pathway operates in platypus (Additional file 1: Figure S1). We conclude that phosphorylation of H2AFX does not occur in platypus pachytene cells.

### Replication protein A (RPA) lacks preferential sex chromosome association in late prophase I spermatocytes

**Fig. 4:**
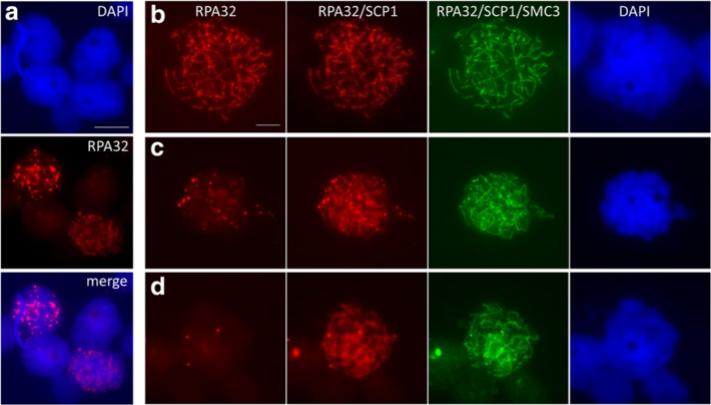
RPA dynamics during platypus prophase I. Platypus testis surface spreads were immunostained with anti-RPA32 followed by serial SYCP1 and SMC3 staining as all three antibodies were raised in rabbit. **a** Abundant RPA32 foci were observed in many nuclei with obvious reductions in nuclei with a large DAPI void typical of cells in advanced stages of prophase I. **b** RPA32 foci decorate all chromosomes prior to synapsis and SC assembly. As prophase I progresses evidenced by SYCP1 element extension and thickening, RPA32 foci numbers are reduced **c** and generally absent by the time SMC3 has heavy accumulation on the sex chromosomes **d**. Scale bar = 5 μm. *DAPI* 4’6-diamidino-2-phenylindole

To further assess meiotic DSB dynamics in platypus prophase I to identify sex chromosome specific accumulations we used an antibody against a replication protein A subunit (RPA32) to visualise the temporal and spatial distribution of DDR foci. RPA functions to stabilise ssDNA ends at sites of SPO11-mediated DSBs during meiotic prophase [30] and specifically persists on unpaired DNA during pachytene [30, 31]. Consistent with observations in mouse, human and chicken [12, 30, 32, 33], platypus meiotic cells have many RPA32 foci decorating the condensing chromosomes in early prophase with an obvious loss of foci in nuclei with a large DAPI negative NLB characteristic of pachytene stage in platypus (Fig. 4a). RPA foci evenly decorate asynaptic chromosomal axes prior to resolution into more restricted and limited domains as SC formation progresses. Previous reports describe persistence of RPA foci specifically on the unsynapsed X and Z of mouse and chicken, respectively, at late pachytene [12, 31, 32]. Therefore, we asked whether such restriction of foci to unpaired sex chromosome DNA also occurs in platypus by assessing their frequency and distribution in late prophase I nuclei. Prophase cells were staged by serial immunostaining with SYCP1 and SMC3 following RPA32 staining (due to all three antibodies being raised in rabbit) and RPA foci numbers and position relative to the NLB were observed. We recently found that the cohesin component SMC3 can be used to visualise the sex chromosome chain in platypus due to its sex chromosome specific heavy loading (Casey et al. personal communication) and, therefore, used this as a meiotic stage and sex chromosome position marker. We did not detect RPA foci distributions indicative of sex chromosome specific accumulation in late prophase I spermatocytes but saw an overall reduction as prophase I progressed (Fig. 4b). No para-NLB RPA foci clusters were observed in SCP1 positive nuclei which also had strong para-NLB SMC3 staining. This indicates an absence of RPA foci persistence on unpaired sex chromosome DNA. Of 138 pachytene nuclei observed, 31 % lacked an RPA signal, 50 % had one to three foci and 8 % had six or more foci, none of which showed linear decorations or para-NLB clustering persisting beyond the stage the autosomal RPA accumulations were lost. We, therefore, do not find any evidence for the presence of ‘lagging’ sex chromosome specific RPA accumulation.

### Platypus prophase I spermatocytes have a reduced general level of transcription

**Fig. 5:**
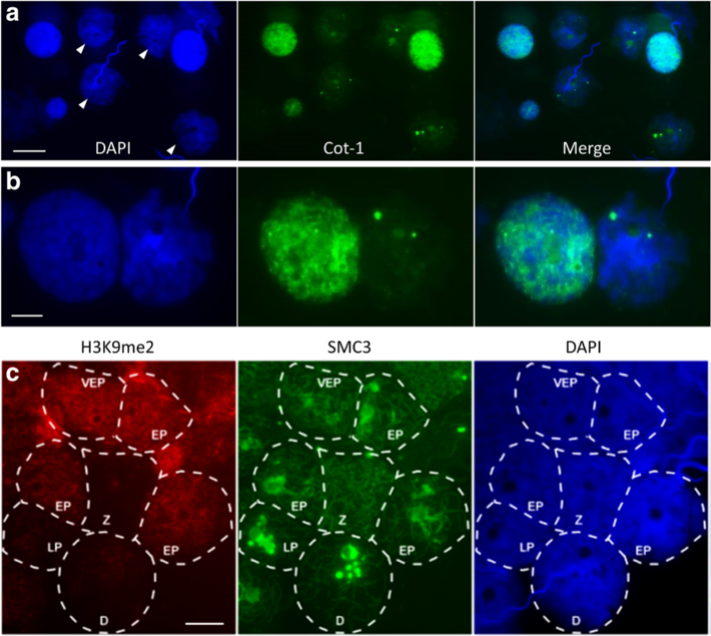
Late prophase I spermatocytes have reduced transcription and increased H3K9me2. Surface spreads of PFA fixed platypus total testis suspensions were hybridised with a Cot-1 RNA FISH probe and counterstained with DAPI or immunostained for H3K9me2 and SMC3. **a** Nuclei with a characteristic DAPI appearance with large DAPI void (*arrowheads*) have a marked reduction in Cot-1 RNA FISH signal while other cell types are positive for Cot-1 hybridisation signal. Scale bar = 20 μm. **b** Note two to three intense Cot-1 foci are consistently present in late prophase I nuclei. Scale bar = 10 μm. **c** H3K9me2 staining was low in zygotene and diplotene but increased through pachytene with reductions in late pachytene (H3K9me2 panel). Z = zygotene, VEP = very early pachytene, EP = early pachytene, LP = late pachytene and D = diplotene. Scale bar = 10 μm. *PFA* paraformaldehyde, *FISH* fluorescence in situ hybridisation, *DAPI* 4’6-diamidino-2-phenylindole

We next asked whether platypus sex chromosomes undergo transcriptional silencing (MSCI) during prophase I. MSCI can be visualised by the absence of Cot-1 RNA FISH signal over the sex body during pachytene in marsupials, mouse and human [34–36]. Surprisingly, we observed striking wholesale reductions in Cot-1 probe hybridisation specifically in prophase I nuclei relative to other cells on PFA fixed whole testis extract surface spreads (Fig. 5a). This indicates global transcriptional reductions occur during the meiotic stage when MSCI is observed in therians. No regions of obvious differential Cot-1 RNA FISH levels were observed in nuclei with low Cot-1 signal with the exception of one to three strong punctate foci (Fig. 5b). This indicates that the transcriptional activity of both autosomes and sex chromosomes were low relative to non-prophase I cells. We then used anti-SMC3 to more accurately stage prophase I nuclei in combination with an antibody against a repressive histone mark (H3K9me2) to further assess the general chromatin status in prophase I nuclei. H3K9me2 staining showed increases in pachytene spermatocytes with reductions prior to (zygotene) and following (late pachytene and diplotene) this stage further supporting the presence of a transient and stage specific period of general transcriptional reduction (Fig. 5c).

### Reduced transcription of autosomal and sex linked genes in prophase I meiotic cells

**Fig. 6:**
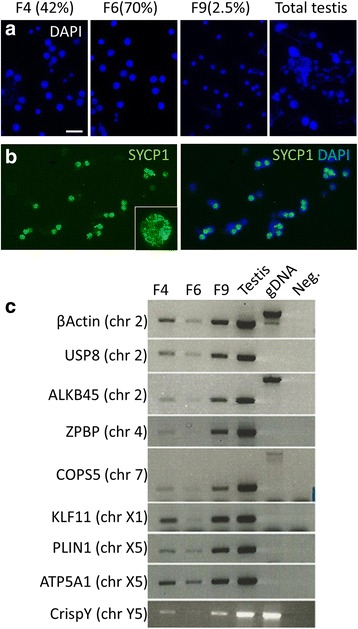
Platypus testis cell separation and gene expression analysis. **a** Total testis cell suspensions were applied to a continuous 2-4 % BSA gradient, fractions eluted and cell populations from each fraction isolated for RNA extraction and RT-PCR. F6 has the highest pachytene enrichment (70 %) followed by F4 (42 %) and F9 (2.5 %). Scale bar = μ20 m. **b** Pachytene or late prophase I fraction content was assessed using surface spreads of each fraction stained for SYCP1. **c** Equivalent cell numbers were used to extract total RNA from each eluted fraction for semi-quantitative RT-PCR. Pachytene enriched fractions have reduced expression of autosomal and sex linked genes. As pachytene content increases the transcript abundance decreases independent of the genes’ genomic location. Controls were total testis from the same animal used for fractions (Testis, *lane 4*), platypus male genomic DNA (gDNA, *lane 5*) and no template (Neg., *lane 6*). Absence of gDNA control is due to primers spanning large intron. Chromosomal location for each gene in parenthesis *left* of panels. *BSA* bovine serum albumin

To further investigate gene transcriptional activity specifically at pachytene we used gravity sedimentation to isolate stage enriched cell populations of at least 70 % pachytene purity assessed by SYCP1 staining surface spreads from each fraction (Fig. 6b and Additional file 2: Figure S2). RNA was extracted from each eluted fraction using equivalent cell numbers and gene specific semi-quantitative RT-PCR was performed using three fractions with markedly different pachytene complements. The three fractions analysed by RT-PCR contained 42 % (F4), 70 % (F6), and 2.5 % (F9) SYCP1 positive cells (Fig. 6a and Additional file 2: Figure S2). Expression analysis and the enrichment profiles are consistent with the observed reductions in Cot-1 RNA FISH signal as we observed corresponding reductions in PCR product abundance specifically in fractions with high SYCP1 positive cell complements (Fig. 6c). The loss of transcript abundance was not biased regarding gene location as autosomal, PAR, and sex chromosome linked genes had similar relative reduction trends in fractions with high SYCP1 positive content compared to fractions with reduced SYCP1 positive complements or total testis (Fig. 6c).

## Discussion

Transcriptional silencing of unpaired DNA during prophase I of meiosis (MSCI) is a conserved mechanism by which the consequence of aberrant synapsis is prevented or heterologous sex chromosomes avoid checkpoint activation [3, 9]. MSCI has been extensively studied in therian mammals and two major papers recently investigated this in chicken with differing conclusions [12, 37]. Monotremes are the third major group of mammals, however, their complex sex chromosome system shares homology to bird sex chromosomes [16–18]. We have therefore used the platypus to determine when mammalian MSCI emerged in evolution. Our data provide first evidence that: 1) MSCI exclusively occurs in therian mammals; 2) similar to birds, monotreme sex chromosome self-association and gene content may circumvent the requirement for MSCI, and 3) nucleolar interactions with sex chromosomes and the future therian X in the basal mammalian lineage may have paved the way toward therian MSCI.

### Platypus sex chromosomes coalesce into a dense mass during prophase I

We identified platypus sex chromosome elements within a compact DAPI intense and SMC3 positive mass reminiscent of the state of synaptic adjustment observed between the ZW during chicken pachynema [15]. Given the absence of detectable DDR components on the platypus sex chromosomes during late prophase I we take this observation and the coalesced sex chromatin in platypus pachytene nuclei to represent a ZW-like state of sex chromosome self-association. We did not observe a SYCP1 positive region within the sex chromatin mass suggesting self-association differs from that occurring in chicken where SC components presumably mediate ZW heterologous synapsis.

Our observation that the platypus sex chromosome chain is tethered to the nucleolus raises questions regarding its contribution to sex chromatin formation. In mouse, nucleoli cease interaction with autosomal NORs in early pachytene and migrate to the sex chromosomes, however, the reason for this interaction remains unclear [21, 22]. Nucleoli contain an abundance of proteins not directly involved in rRNA production [38] and sex body association implicates a chromatin modifying role in mouse MSCI. Similarly, we show loss of ribosomal nucleolar activity during the period of physical attachment to the condensed sex chromosomes and speculate an involvement in establishing a localised chromatin modifying environment. We also report here that platypus chromosome 6 (which contains the only NOR), while maintaining restricted association with the NLB during late prophase I, also shows H3K9me2 at the region of association on the p arm where the rDNA is located. As yet the significance of these observations remains unclear but it is intriguing given that monotreme chromosome 6 represents the future therian X [17].

### Platypus sex chromosomes and autosomes both maintain a state of reduced transcription in SYCP1 positive late prophase I spermatocytes

**Fig. 7:**
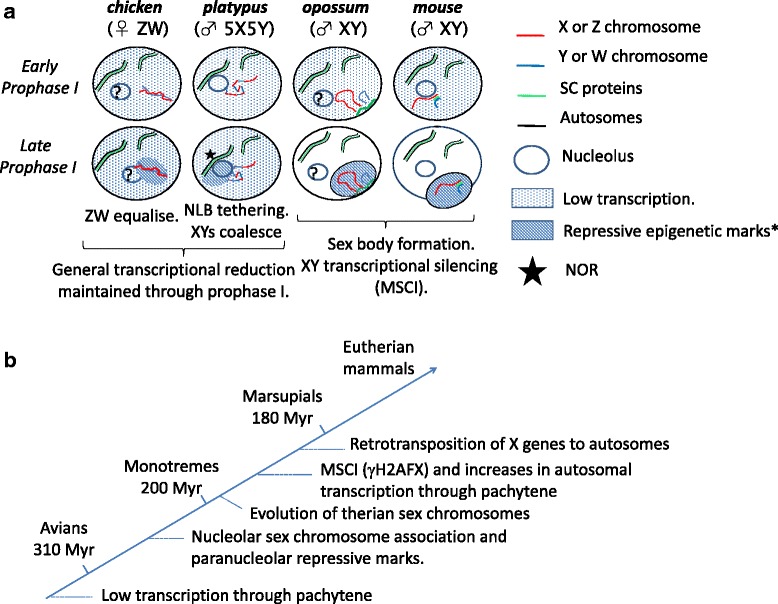
Comparison of amniote sex chromosome systems through prophase I and evolutionary pathway to mammalian MSCI. **a** Chicken and platypus are grouped due to lack of X borne retrogenes, ZW/XY self-association dynamics, low general states of late prophase I transcription and absence of sex body. Nucleolar tethering of the platypus sex chromosome chain and chromosome 6 are prominent features in late prophase I spermatocytes along with para-nucleolar repressive epigenetic mark associations. Question mark in nucleolus denotes unknown prophase I dynamics in chicken and marsupials. * Repressive epigenetic marks on the chicken ZW at late pachytene are restricted to constitutive heterochromatin and proposed to not be part of a MSCI pathway [12]. **b** Following monotreme divergence the mammalian lineage utilised a different sex chromosome system leading to MSCI. The emergence of therian MSCI involved a change from general low states of transcription levels through prophase I to sex chromosome specific silencing. This triggered the movement of X genes to autosomes via retrotransposition. The monotreme sex chromosome complex nucleolar tethering and persistence of chromosome 6 nucleolar association in combination with the establishment of paranucleolar repressive machinery may have been the precursors then recruited into the new therian sex chromosome MSCI system

We have assessed the transcriptional activity of meiotic prophase cells in platypus males using Cot-1 RNA FISH, cell enrichment, and RT-PCR to determine the status of sex chromosome specific transcriptional silencing. Unlike observations in mouse describing early prophase I transcriptional reduction followed by selective autosomal transcription increases during pachytene [39, 40], we observe expression reductions occurring and being maintained through late prophase I for both autosomal and sex chromosome linked genes. In addition, we fail to detect any regions within Cot-1 RNA FISH stained late prophase I nuclei that show obvious differences at late prophase I. Therefore, our data provide no evidence for differential transcriptional activity between the synapsed autosomes and the sex chromosome chain in pachytene spermatocytes which represents a major departure from the therian MSCI programme (summarised in Fig. 7).

### Platypus sex chromosomes lack specific repressive histone mark accumulations observed on the therian sex body

The large H2A histone variant H2AFY is associated with both the inactive X chromosome in female somatic cells and the therian XY sex body [27–29, 41]. In addition, more specific roles, such as strengthening sex chromosome PAR associations and sister chromatid cohesion to aid correct segregation, have been put forward [42, 43]. Our observation that H2AFY accumulates specifically at pachytene and is only transiently associated with selected sex chromosomes is unlike the sex body specific H2AFY accumulation observed in mammals [28, 29]. Possibly in platypus this represents NOR associated accumulations of a repressive histone variant which is then co-opted into the therian MSCI pathway. Sex chromosome self-synapsis appears to prevent wholesale recruitment of DDR machinery not only in avians and platypus but also therians. It has been demonstrated that sex chromosome self-synapsis circumvents a silencing response in XO female mice as the single X fails to accumulate BRCA1, γH2AFX or ATR when self-associated [44]. This phenomenon has also recently been observed to occur in domestic dog meiosis [45]. Interestingly, our observed persistent NLB association with the only NOR in platypus on chromosome 6 may also implicate a role for H2AFY in negatively regulating rDNA transcriptional activity as recently described [46]. In addition to X chromosome inactivation the nucleolus is a key regulator of nuclear-wide heterochromatin organisation and inheritance and more recently stem cell differentiation programmes [47–49]. The H3K27me3 and H3K9me3 accumulations are unexpected and require additional colocalisation studies to explore their significance. Therefore, we find no evidence to implicate these repressive marks in a sex chromosome specific silencing role. The close nucleolar association of platypus chromosome 6 when rRNA biogenesis has ceased may indicate therian sex body formation originated from initial colocalisations with nucleolar repressive chromatin domains present in the basal mammalian lineage.

### Platypus sex chromosomes lack a therian-like MSCI programme

Unsynapsed autosomes and unpaired XY DNA at pachytene triggers DDR machinery accumulation to protect the cell from meiotic progression until synaptic completion or to escape checkpoint surveillance, respectively [44, 50]. We used anti-RPA32 to determine whether platypus unpaired sex chromosome DNA accumulates repair pathway components indicative of meiotic silencing. We hypothesised two scenarios: firstly our observations that platypus spermatocytes undergo a global phase of reduced transcription through pachytene when the sex chromosomes have extensive self-association resembles recent observations in chicken [12, 37]. Secondly, the presence of unpaired DNA would trigger MSCI and we would detect DDR component accumulation on heterologous sequences similar to mammals and the asynaptic ZW [12]. RPA foci dynamics demonstrate that the genome-wide first wave of DNA DSBs in early prophase is present in platypus.

If a therian-like response to unpaired DNA operated in platypus meiosis, RPA persistence specifically on sex chromosomes should be observed. We were unable to identify RPA foci patterns consistent with a persistence of sex chromosome specific RPA accumulation. The fact that we do not observe any late prophase I (SYCP1 positive) nuclei with clustered RPA foci configurations suggests platypus sex chromosome self-association is remarkably efficient and precludes involvement of key meiotic checkpoint machinery. We, therefore, find no evidence to support the idea that sex chromosome DNA triggers discreet checkpoint pathway responses and sex chromosome self-association may preclude a mammalian MSCI response.

A recent report on the presence of X-borne retrogenes postulated the emergence of MSCI occurring prior to the metatherian-eutherian split and after monotreme divergence based on their absence in platypus and chicken [7]. To better understand these evolutionary transitions it would be informative to assess rRNA transcription and sex chromosome-nucleolar interactions in female chicken meiosis to determine whether this is unique to platypus or the ancestral state prior to therian MSCI and X inactivation.

## Conclusions

Our data support the idea that a therian-like MSCI checkpoint does not operate in platypus. We show close association of multiple repressive histone marks with the nucleolus and speculate proximity to a transcriptionally repressive perinucleolar region was the first step towards sex chromosome specific silencing. Originally established to regulate NOR activity through pachytene, this repressive perinucleolar environment possibly has then been recruited into the MSCI pathway (Fig. 7b).

## Materials and methods

### Sample collection

Platypus were captured at the Upper Barnard River (New South Wales, Australia) under the following permits: AEEC permit R.CG.07.03 (F.G.), Environment ACT permit LI 2002 270 (J.A.M.G.), NPWS permit A193 (R.C.J.), and AEC permit no. S-49-2006 (F.G.). Animals were euthanized with an intraperitoneal injection of 0.1 mg/g pentobarbital (Lethabarb, ​VIRBAC Pty. Ltd., Australia).

### Gravity sedimentation

Total testis cell lysates were stored in 90 % phosphate-buffered saline (PBS)/10 % dimethyl sulfoxide (DMSO) at −80 °C. A total of 500 μL of total testis suspension was diluted in 9.5 ml ice cold PBS, filtered through a 70 μm cell strainer (Falcon, #2530), pelleted at 700 rpm for 5 min and resuspended in 10 ml ice cold RPMI media 1640 (Life Technologies, Carlsbad, CA, USA ). A 2-4 % continuous bovine serum albumin gradient was formed using a 100 ml total volume dual chamber SG100 gradient maker (Hoefer, Inc. Holliston, MA, USA). in a 250 ml Lenz Bistabil separating funnel (Lenz Laborglas GmbH & Co.KG, Wertheim, Germany). Sedimentation was for 3.5 hours 10 x 10 ml fractions decanted from the funnel. Cell fractions were used for surface spreading and RNA extraction.

### Antibodies and BAC clones

The following antibodies were used for surface spreading and fibroblast immunohistochemistry at 1:300 dilution. Anti- H2AFY rabbit (Merck Millipore, Victoria, Australia), − SYCP1 rabbit (Novus Biologicals Littleton, CO 80120, USA), − SMC3 rabbit (Abcam, Cambridge, UK), − γH2AFX rabbit (Abcam, Cambridge, UK ), −H3K9me2 mouse (Abcam, Cambridge, UK), −H3K9me3 mouse (Abcam, Cambridge, UK), −Fibrillarin mouse (Abcam, Cambridge, UK), −ATM phospho S1981 mouse (Abcam, Cambridge, UK), −Histone H4 rabbit (Merck Millipore, Victoria, Australia), −RNA PolII mouse (Merck Millipore, Victoria, Australia) and -RPA32 rabbit (Abcam, Cambridge, UK).

BAC probes were obtained from Children’s Hospital Oakland Research Institute (CHORI, Oakland, CA, USA) or CUGI BAC/EST Resource Centre, Clemson, SC, USA: X1q (CH236-378 F21), PAR1 (CH236-286 H10), PAR3 (CH236-78 K11), PAR4 (CH236-165 F5), PAR5 (CH236-462 C1), PAR6 (CH236-639 O23), PAR8 (OA_Bb-466 A15), Y2 (CH236-178 N20), Y5 (CH236-152 P15) and X5 (CH236-236 A5).

### Semi-quantitative RT-PCR

Equivalent cell numbers from each gravity sedimentation elution fraction were determined using a haemocytometer followed by standard TriZol (Life Technologies, Carlsbad, CA, USA) RNA extraction according to the manufacturers’ instructions. A sample of 130 ng of total RNA was used as template for cDNA synthesis using SuperScript III Reverse Transcriptase (Life Technologies, Carlsbad, CA, USA).

### Image acquisition

All DNA FISH and immunostaining signals were observed using a Zeiss Axio-Imager 2.1 microscope with 10x ocular and 10x, 20x, 63x, and 100x objective lenses. Images were captured using an Axiocam CCD camera and processed with Zeiss Axiovision or ImageJ (NIH, USA) software.

### Sample preparation

For DNA FISH followed by immunofluorescence, testis lysates were thawed, rinsed in 1x PBS and resuspended in 0.1 M sucrose, incubated on ice for 5 min before being applied to microscope slides. Slides were then flooded with 4 % PFA (pH 7.6), incubated in a humid chamber for 2 hrs at room temperature prior to rinsing in water and air drying. For DNA FISH only, testis lysates were fixed in 3:1 methanol:acetic acid, dropped onto a humidified microscope slide and air dried.

### DNA fluorescence in situ hybridisation and immunofluorescence

#### DNA FISH

Fluorescent probes were generated by random labelling 1 μg of template BAC DNA overnight using Spectrin orange- or green-dUTP followed by precipitation with salmon sperm and sonicated male platypus genomic DNA. Pellets were resuspended in 50 % formamide/50 % 2x dextran sulfate hype buffer. Slides were dehydrated, RNAseA and pepsin treated, fixed in 1 % formaldehyde before dehydration and denaturing in 50 % formamide at 70 °C. Probes were incubated overnight in a humid chamber at 37 °C. Slides were washed 3x 5 min in 50 % formamide/2x SCC at 42 °C, 0.1x SCC at 60 °C, DAPI stained and mounted (Vectashield, Vector Laboratories, Burlingame, CA, USA).

#### Immunofluorescence

PFA fixed meiotic spread slides were blocked (1xPBS/0.5 % BSA/0.5 % milk powder) 3x5min prior to primary antibody incubation in 1xPBS/10 % BSA, washed 3x5min in 1xPBS, 3x5min in 1xPBS/10 % goat serum/5 % milk powder prior to secondary antibody (1:500) incubation. Slides were washed 3x5min in 1xPBS, DAPI stained and mounted (Vectashield, Vector Laboratories USA).

### Cot-1 RNA Fluorescence in situ Hybridisation

Frozen testis was shaved into a suspension of 1xPBS which was then dropped onto slides. Cells were permeabilised with ice cold CSK buffer (100 mM NaCl, 300 mM sucrose, 3 mM MgCl, 10 mM PIPES supplemented with 0.5 % Triton X-100 and 2 mM Vanadyl Ribonucleoside before use) on an ice cold platform for 10 min. Slides were drained and fixed in ice cold freshly made 4 % PFA for 10 min then rinsed in ice cold 1xPBS. Slides were then dehydrated using a series of ice cold ethanol and air dried. Platypus Cot-1 DNA (1 μg) was randomly labelled with Spectrin green-dUTP followed by precipitation with salmon sperm. Pellets were resuspended in RNAse free 50 % formamide/50 % 2x dextran sulphate hybe buffer, denatured and applied to slides and incubated overnight in a humid chamber at 37 °C. Slides were washed 3 x 5 min in 42 °C 50 % formamide/1x SSC, 3 x 5 min 2x SSC at room temperature then mounted in Vectorshield with DAPI.

### Fibroblast irradiation

Primary fibroblasts from mouse and platypus were cultured on microscope slides overnight in 50 % DMEM/50 % Amniomax media prior to exposure to 5 Grey of ionizing radiation using the blood sterilisation facility at the Royal Adelaide Hospital. Slides were incubated post treatment in media for an additional 90 min before a PBS wash, 0.15 % triton/PBS permeabilisation and 4 % PFA fixation for immunostaining as above.

## Additional files

Additional file 1: Figure S1. DNA DSB induction in mouse and platypus fibroblasts. Fibroblasts grown on slides were exposed to 5 Grey of ionising radiation, PFA fixed and dual immunostained for phosphorylated H2AFX (γH2AFX) and ATM after 1.5 hours to detect double strand break repair foci. Marked induction of DNA breaks was observed and both antibodies showed signal co-localisation demonstrating the ability of the γH2AFX antibody to detect DSBs in both platypus and mouse. Scale bar = 10 μm. (PNG 3328 kb) Additional file 2: Figure S2. Platypus prophase I cell enrichment by gravity sedimentation. Total testis cell suspensions were applied to a continuous 2-4 % BSA gradient, fractions eluted and cells from each fraction harvested for surface spreading. Gradient fractions are numbered as they were eluted from the sedimentation chamber, fields from post elution fraction spreads shown at 20x and 60x magnification. F6 has the highest pachytene enrichment (70 %) followed by F4 (42 %) and F9 (2.5 %). (PNG 2371 kb)
