# Supplementary figures and images for: Lack of sex chromosome specific meiotic silencing in platypus reveals origin of MSCI in therian mammals
Source: BMC Biol. 2015 Dec 10;13:106. doi: 10.1186/s12915-015-0215-4 (PMC4676107; doi:10.1186/s12915-015-0215-4)

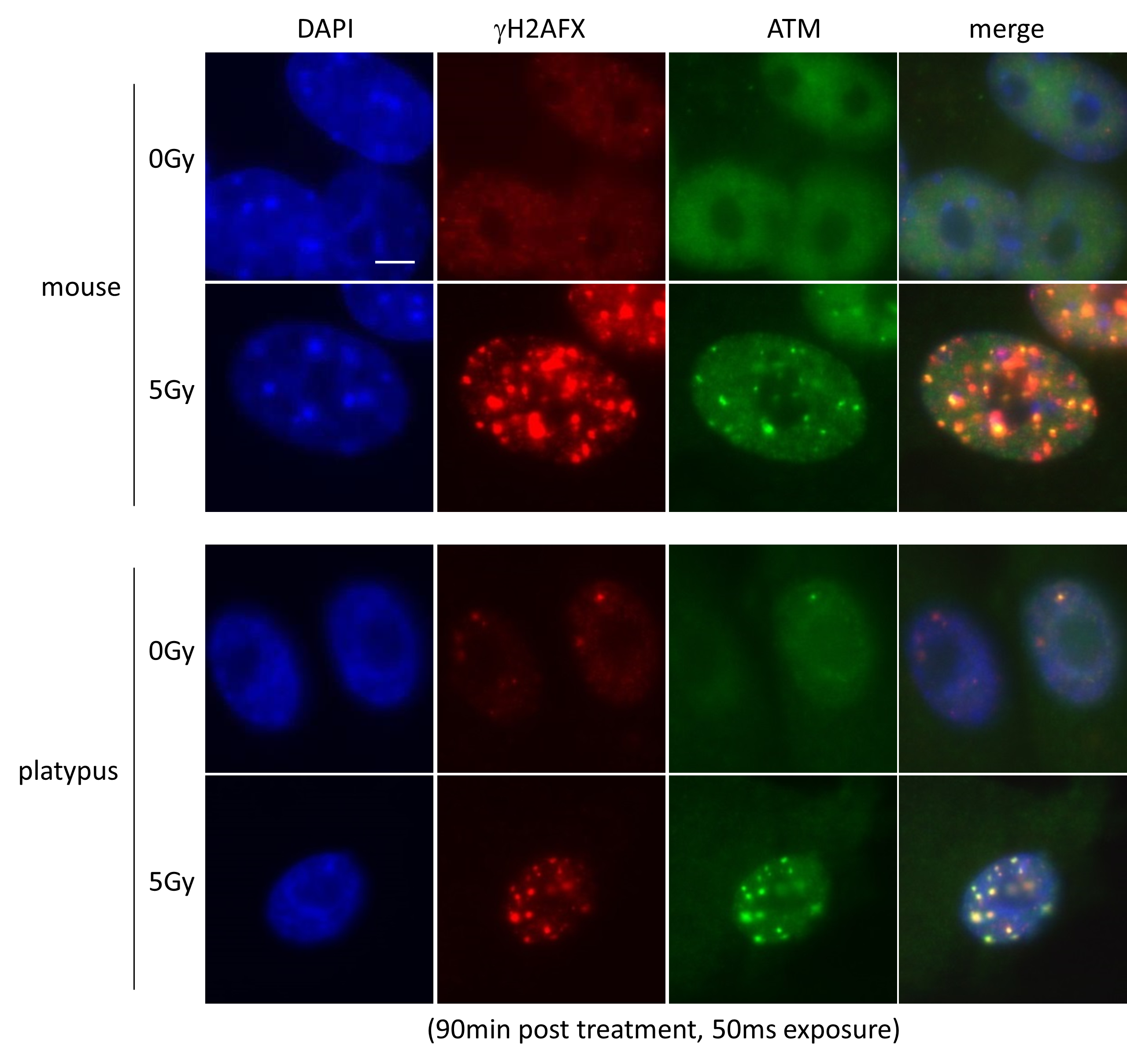

Supplement: Additional file 1: Figure S1. — DNA DSB induction in mouse and platypus fibroblasts. Fibroblasts grown on slides were exposed to 5 Grey of ionising radiation, PFA fixed and dual immunostained for phosphorylated H2AFX (γH2AFX) and ATM after 1.5 hours to detect double strand break repair foci. Marked induction of DNA breaks was observed and both antibodies showed signal co-localisation demonstrating the ability of the γH2AFX antibody to detect DSBs in both platypus and mouse. Scale bar = 10 μm. (PNG 3328 kb) [file 12915_2015_215_MOESM1_ESM.png]

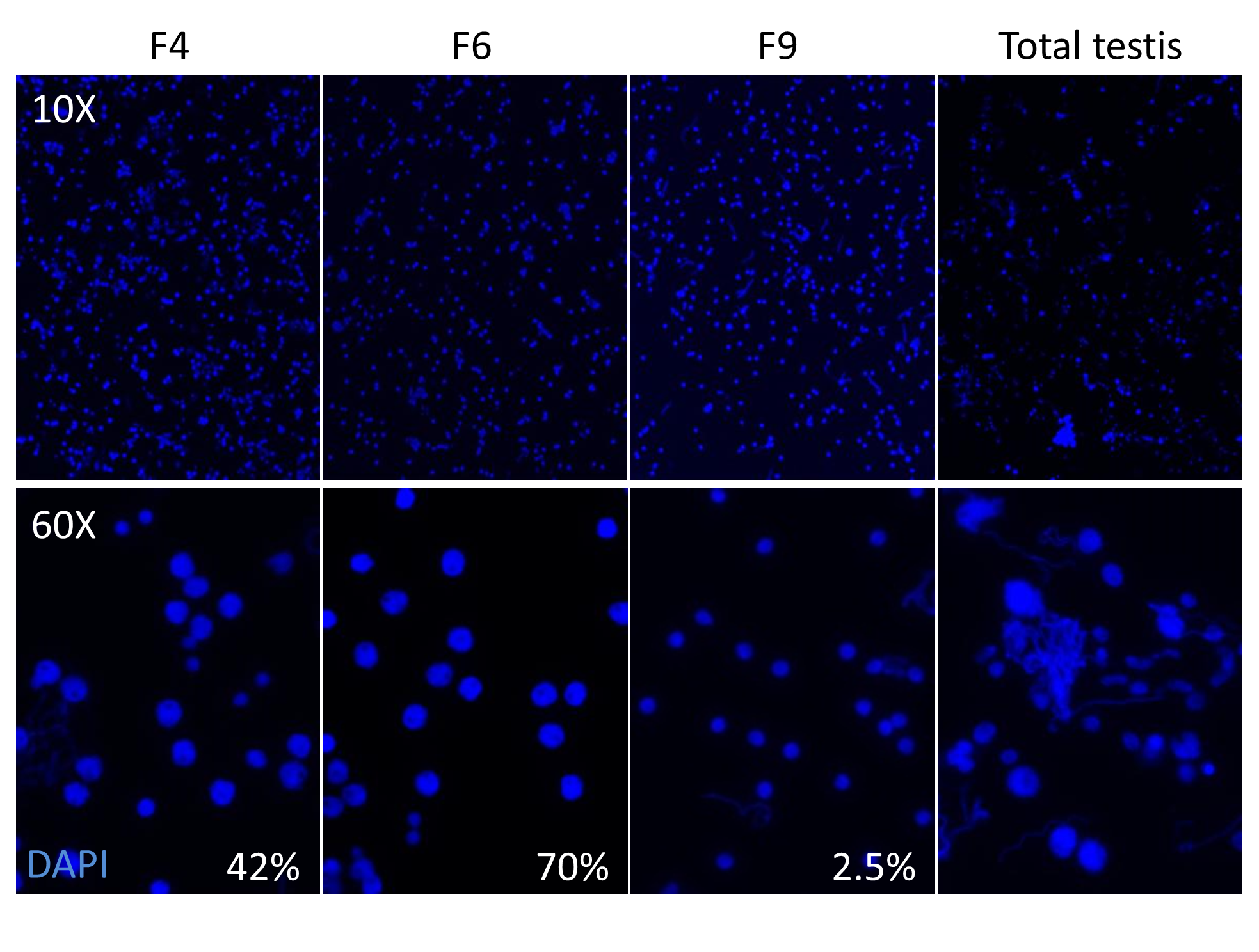

Supplement: Additional file 2: Figure S2. — Platypus prophase I cell enrichment by gravity sedimentation. Total testis cell suspensions were applied to a continuous 2-4 % BSA gradient, fractions eluted and cells from each fraction harvested for surface spreading. Gradient fractions are numbered as they were eluted from the sedimentation chamber, fields from post elution fraction spreads shown at 20x and 60x magnification. F6 has the highest pachytene enrichment (70 %) followed by F4 (42 %) and F9 (2.5 %). (PNG 2371 kb) [file 12915_2015_215_MOESM2_ESM.png]
